# Identification and Spatiotemporal Expression of a Putative New GABA Receptor Subunit in the Human Body Louse *Pediculus humanus humanus*

**DOI:** 10.3390/genes15070844

**Published:** 2024-06-27

**Authors:** Omar Hashim, Berthine Toubaté, Claude L. Charvet, Aimun A. E. Ahmed, Cédric Neveu, Isabelle Dimier-Poisson, Françoise Debierre-Grockiego, Catherine Dupuy

**Affiliations:** 1 BioMAP, UMR ISP 1282 INRAe, Université de Tours, 37200 Tours, France; omar.hashim@univ-tours.fr (O.H.); berthine.toubate@univ-tours.fr (B.T.); isabelle.poisson@univ-tours.fr (I.D.-P.); francoise.debierre@univ-tours.fr (F.D.-G.); 2 Department of Pharmacology, University of Gezira, Wad Madani 21111, Sudan; 3 MPN, UMR ISP 1282 INRAe, Université de Tours, 37380 Nouzilly, France; claude.charvet@inrae.fr (C.L.C.); cedric.neveu@inrae.fr (C.N.); 4 Department of Pharmacology, Albaha University, Al Baha 65799, Saudi Arabia; aimun725@hotmail.com; 5 Department of Pharmacology and Toxicology, Omdurman Islamic University, Omdurman 14415, Sudan

**Keywords:** *Pediculus humanus humanus*, human body louse, GABA receptors, HoCas

## Abstract

The human louse (*Pediculus humanus*) is an obligatory blood feeding ectoparasite with two ecotypes: the human body louse (*Pediculus humanus humanus*), a competent vector of several bacterial pathogens, and the human head louse (*Pediculus humanus capitis*), responsible for pediculosis and affecting millions of people around the globe. GABA (γ-aminobutyric acid) receptors, members of the cys-loop ligand gated ion channel superfamily, are among the main pharmacological targets for insecticides. In insects, there are four subunits of GABA receptors: resistant-to-dieldrin (RDL), glycin-like receptor of drosophila (GRD), ligand-gated chloride channel homologue3 (LCCH3), and 8916 are well described and form distinct phylogenetic clades revealing orthologous relationships. Our previous studies in the human body louse confirmed that subunits Phh-RDL, Phh-GRD, and Phh-LCCH3 are well clustered in their corresponding clades. In the present work, we cloned and characterized a putative new GABA receptor subunit in the human body louse that we named HoCas, for Homologous to Cys-loop α like subunit. Extending our analysis to arthropods, HoCas was found to be conserved and clustered in a new (fifth) phylogenetic clade. Interestingly, the gene encoding this subunit is ancestral and has been lost in some insect orders. Compared to the other studied GABA receptor subunits, HoCas exhibited a relatively higher expression level in all development stages and in different tissues of human body louse. These findings improved our understanding of the complex nature of GABA receptors in *Pediculus humanus* and more generally in arthropods.

## 1. Introduction

The human louse *Pediculus humanus* is an obligatory blood feeding ectoparasite. Two known ecotypes of human louse exist: the head louse *Pediculus humanus capitis* and the body louse *Pediculus humanus humanus* (Phh) [1,2]. Currently, pediculosis due to head louse infestations remains one of the most prevalent parasitic infestation in humans, essentially due to the misuse of non-chemical products (silicon-based formulations) and development of resistance to commonly used pediculicides [3]. The human body louse is a known vector of three bacterial pathogens: *Rickettsia prowazekii*, responsible for epidemic typhus [4,5]; *Borrelia recurrentis*, responsible for relapsing fever; and *Bartonella quintana*, which causes trench fever [6]. In the past, massive epidemics of relapsing fever have affected Africa and Eurasia, and recently many cases of louse-borne relapsing fever have been diagnosed in Germany [7], the Netherlands [8], Switzerland [9], Finland [10], and Italy [11].

GABA (γ-aminobutyric acid) receptors are members of the cys-loop ligand gated ion channels (cys-loop LGICs) superfamily [12,13]. They share common molecular features: the cys-loop located in the extra cellular N-terminal domain, four transmembrane domains (TM1-TM4) architecting the body of the channel, and a large highly variable intracellular domain regulating the channel functions and containing most of the protein activation sites.

In insects, GABA receptors play an important role in the transmission of impulses in the central nervous system and neuromuscular junctions that makes them suitable targets for insecticides [14]. Moreover, many physiological functions of GABA receptors like the regulation of central pattern generators and the modulation of information in higher brain processing centers have been described [15]. In the honey bee *Apis mellifera*, picrotoxin and fipronil, known GABA receptor antagonists, impaired olfactory discrimination and sucrose perception, respectively [13].

Four subunits of GABA are described in insects, and at the phylogenetic level, these subunits form four distinct clades revealing orthologous relationships: resistant-to-dieldrin (RDL), glycin-like receptor of drosophila (GRD), ligand gated chloride channel homologue3 (LCCH3), and 8916 [16,17,18,19,20,21].

The first identified insect GABA receptor subunit was RDL in the fruit fly *Drosophila melanogaster*, which forms an anionic inhibitory homo-pentameric receptor [16]. Then, RDL was characterized in many other insects [20,22,23]. LCCH3 and GRD subunits were cloned and described first in *D. melanogaster* [24,25]; GRD/LCCH3 hetero-pentameric receptors capable of forming cationic channel were characterized in different arthropods, including *D. melanogaster* [26], the mite *Varroa destructor* [27], and *A. mellifera* [28]. Moreover, genes encoding 8916 have been cloned in different insect species, including *A. mellifera* [13], *D. melanogaster* [12], *Laodelphax striatellus* [19], and *Chilo suppressalis* [29].

Our previous exploration of GABA receptor subunits in the human body louse *Pediculus humanus humanus* revealed that the genome encodes for three GABA receptor subunits, Phh-RDL, Phh-GRD, and Phh-LCCH3, that are expressed throughout the developmental stages and in different tissues [30]. Moreover, Phh-RDL was able to reconstitute anion-selective functional homomeric receptor while Phh-GRD and Phh-LCCH3 combined to form cation-selective hetero-pentameric receptors [30,31].

Interestingly, besides the four well-described GABA receptor subunits, an additional gene closely related to GRD and 8916 has been described in the German cockroach *Blattella germanica* (Bg-8916_2) and the American cockroach *Periplaneta americana* (Pa-8916_2) [18] as well as in the Chelicerata species, the European sheep tick *Ixodes ricinus* (Ir-GABA3), the common house spider *Parasteatoda tepidarorium* (Pt-GABA3), and the western predatory mite *Galendromus occidentalis* (Go-GABA3) [32].

In the present study, our analysis in the genome of the human body louse allowed us to identify a homolog of these genes annotated as *γ-aminobutyric-acid receptor α-2 subunit precursor* (PHUM507160; XM_002430955.1). Based on a phylogenetic analysis among other GABA receptor subunits, this gene was found to form a new (fifth) clade. We renamed the newly identified gene *hocas* for Homologous to Cys-loop α-like subunit.

## 2. Materials and Methods

### 2.1. Insects

Strain of the human body louse *Pediculus humanus humanus* was reared at BioMAP laboratory (University of Tours, France), maintained in standard conditions (temperature 30 °C, relative humidity 60–70%) and fed on rabbit blood four times per week. This strain of human louse was never exposed to chemical compounds.

### 2.2. RNA Extraction and cDNA Synthesis

Total RNA was extracted from 30 mg of young adult lice, nits, and larvae (L1, L2, L3), as well as the heads, thoraxes, and abdomens of adult lice using RNA plus extraction kit (Machery Nagel, Hoerdt, France) as per manufacturer’s instructions. The cDNA was synthesized by reverse transcription from 1 mg of RNA using superscript III reverse transcriptase (Invitrogen^TM^, Thermo Fisher Scientific, Courtaboeuf, France) using 0.5 mM gene specific primer and 1.5 mM oligo dT primer according to manufacturer’s instructions.

### 2.3. Sequence Analysis and Phylogeny

Using BLASTN, we used the genomic sequences of *Blattella germanica* (Bg-8916_2), *Periplaneta americana* (Pa-8916_2), *Ixodes ricinus* (Ir-GABA3), *Parasteatoda tepidarorium* (Pt-GABA3), and *Galendromus occidentalis* (Go-GABA3) to search for homologues in the genome of the human body louse *Pediculus humanus humanus* from Vector Base^®^ [33]. For sequence analysis, all cloned transcripts were compared with the putative sequences of *P. humanus* deposited in Vector Base^®^ (https://vectorbase.org/vectorbase/app, accessed on 20 May 2022) using Geneious software (Biomatters, Auckland, New Zealand, license number 1ADA-16DF-FE93-DO6-3BF1) and the Basic Local Alignment Search Tool (BLAST^®^, U.S. National Library of Medicine, https://blast.ncbi.nlm.nih.gov/Blast.cgi, accessed on 20 May 2022). Amino acid sequences deduced from full-length transcripts were obtained from ExPASy translate (Swiss Institute of Bioinformatics, https://web.expasy.org/translate/, accessed on 1 July 2022), signal peptide cleavage sites were predicted using the SignalP-5.0 server (Centre for Biological Sequence Analysis, http://www.cbs.dtu.dk/services/SignalP/, accessed on 8 May 2022) [34], and the transmembrane domains were identified using the TMHMM program (http://www.cbs.dtu.dk/services/TMHMM/, accessed on 15 August 2022).

Multiple sequence alignments were carried out by clustal omega algorithm [35] and then viewed and annotated by Jalview software (https://www.jalview.org/, accessed on 1 June 2022) with the sequences of *Laodelphax striatellus* Ls-alphalike (RZF33096.1), *B. germanica* Bg-8916_2 (QQH14694.1), *P. americana* Pa-8916_2 (QQH14658.1), *I. ricinus* Ir-GABA3 (UOV21278.1), *G. occidentalis* Go-GABA-3 (XP_028968418.1), and *P. tepidarorium* Pt-GABA3 (XP_015925419.1).

The phylogenetic trees were constructed in Molecular Evolutionary Genetics Analysis (MEGAXI) [36] using the neighbour-joining (Poisson model) or maximum likelihood methods based on the model of Whelan and Goldman [37], the best model being assessed with MEGAXI, and branch support being assessed with 1000 bootstrap replications.

The trees were constructed with the sequences of RDL, GRD, LCCH3, 8916, α-like, and GABA3 of the insects Collembola, Crustacea, and Chelicerata, obtained from the National Centre for Biotechnology Information (NCBI) database (https://www.ncbi.nlm.nih.gov, accessed on 20 September 2022); accession numbers of sequences were indicated in Figures 2 and 3.

### 2.4. RACE-PCR and Cloning of Full Length Transcripts

**Figure 1:**
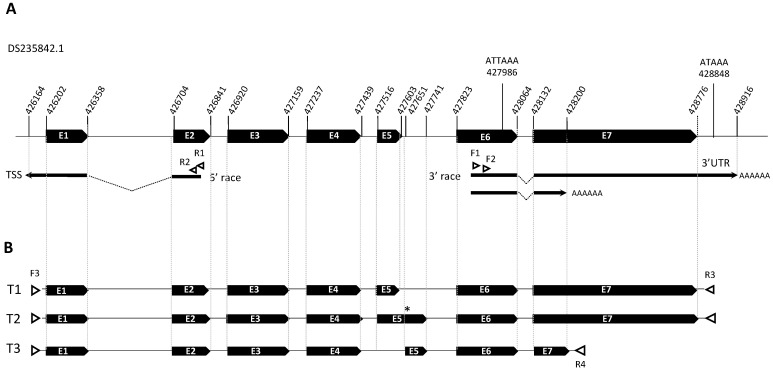
Genomic and transcript organizations of *Phh-hocas*. Exons are represented by black boxes in expanded views of the contigs DS235842.1 with the annotated coding sequence ((**A**), top drawing) and transcripts (**B**). Positions of the primers are indicated by triangles; 5′ and 3′ extremities are indicated by black arrows. The bottom drawings (**B**) show the organization of the 3 cloned *Phh-HoCas* transcripts. * premature stop codon.

The 5′ ends and 3′ ends of *γ-aminobutyric-acid receptor α-2 subunit precursor* (PHUM507160; XM_002430955.1) were characterized by rapid amplification of cDNA ends (RACE-PCR) using a Gene Racer^®^ kit (Invitrogen, Thermo Fisher Scientific, Courtaboeuf, France) as previously described [30] using gene-specific and nested specific primers (Figure 1, Table 1). Cycles for the first and second nested PCR were 94 °C for 5 min and then 35 cycles of 94 °C for 30 s, 58 °C for 30 s, 72 °C for 1 min, and final extension 72 °C for 5 min. Full-length *γ-aminobutyric-acid receptor α-2 subunit precursor* (PHUM507160; XM_002430955.1) was amplified using 1 µL of cDNA and 10 pmol of two gene specific primers (Table 1) using GoTaq DNA Polymerase (Promega, Charbonnières les Bains, France) according to the manufacturer’s instructions. After migration on agarose gel, the PCR products were purified using Nucleo Spin Gel and a PCR clean-up kit (Macherey-Nagel, Hoerdt, France) according to the manufacturer’s instructions. RACE-PCR and RT-PCR products were cloned in PGEM-T Easy vector (Promega) and sequenced by Eurofins genomics.

### 2.5. Spatial and Temporal Expression

Quantitative PCR (qPCR) analysis was performed on 100 ng of RNA extracted from adult lice; nits; and L1, L2, and L3 larval stages, as well from heads, thoraxes, and abdomens of adult lice as described in section “RNA extraction, cDNA synthesis and RACE-PCR” using the primers listed (Table 2). A 100 ng of RNA was added to 10 µL of Sybergreen master mix (Thermo Fisher Scientific) and 10 pmol of gene-specific primers (Table 2) for a final volume of 20 µL. The qPCR was performed with primary denaturation at 95 °C for 10 min followed by 40 cycles of 95 °C for 15 s and 60 °C for 1 min using the StepOnePlus Real-time PCR System (Applied Biosystems, Thermo Fisher Scientific) following the conditions recommended by the manufacturer and by applying comparative cycle threshold experiment 2^−(ΔCt)^ with *β-actin* as an endogenous control. The data from resulting relative expressions were analyzed by ANOVA test followed by Tukey’s multiple comparisons test and plotted as median using GraphPad Prism 7.

## 3. Results

### 3.1. Phylogenetic Analysis and Sequence Identity

Using the mRNA sequences of *Blattella germanica* (Bg-8916_2), *Periplaneta americana* (Pa-8916_2), *I. ricinus* (Ir-GABA3), *P. tepidarorium* (Pt-GABA3), and *G. occidentalis* (Go-GABA3) in BLASTN analysis against the genome of the human body louse *Pediculus humanus humanus* (Vector Base^®^), we retrieved a putative *γ-aminobutyric-acid receptor α-2* subunit precursor gene (PHUM507160; XM_002430955.1). The genomic sequence of PHUM507160 contains 2575 bp organized in 7 exons located in the super contig DS235842.1 (Figure 1A).

**Figure 2:**
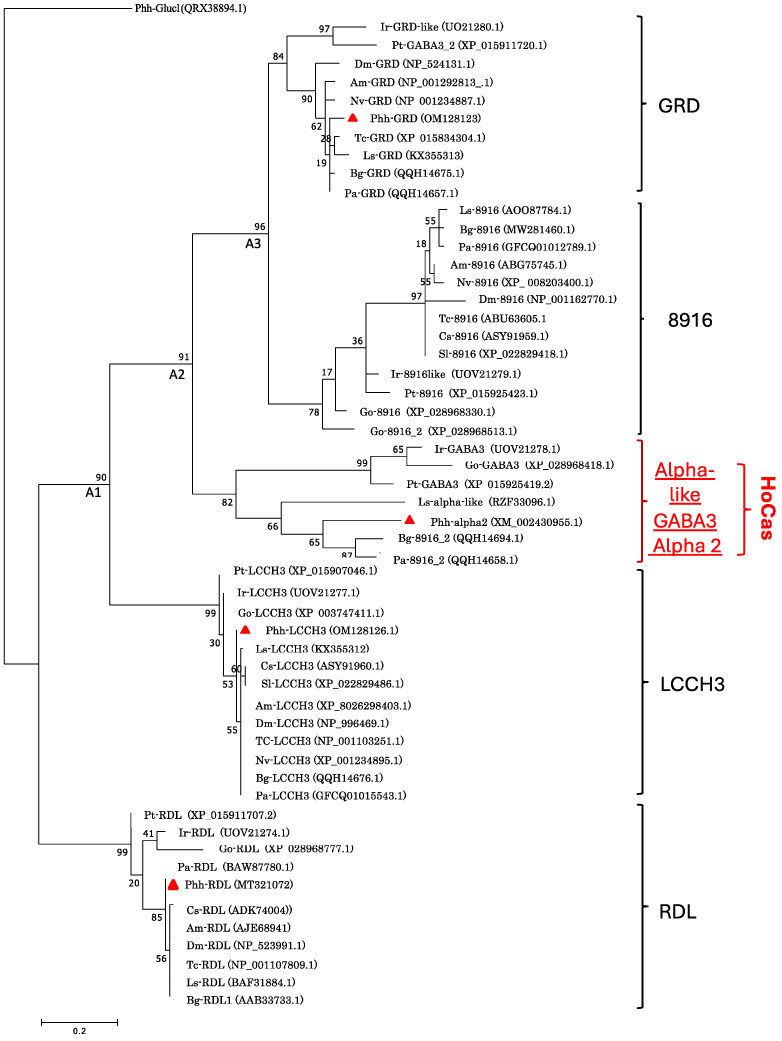
Phylogenic tree of Phh-GABA receptor subunits compared with those of other arthropod species. The phylogenic tree was constructed by maximum likelihood methods based on Whelan and Goldman, the best model being assessed with MEGAXI, and branch support being assessed with 1000 bootstrap replications. *P. humanus* sequences are labeled with red triangles. A1: common ancestor of LCCH3, HoCas, 8916 and GRD. A2: common ancestor of HoCas, 8916 and GRD. A3: common ancestor of 8916 and GRD. Am for *Apis mellifera;* Bg for *Blatella germanica;* Dm for *Drosophila melanogaster;* Cs for *Chilo suppressalis;* Go for *Galendromus occidentalis;* Ir for *Ixodes ricinus;* Ls for *laodelphax striatellus;* Nv for *Nasonia-vitripenis;* Pa for *Periplaneta americana;* Phh for *Pediculus humanus humanus;* Pt for *Parasteatoda tepidarorium;* Sl *for Spodoptera litura;* Tc for *Tribolium castaneum*.

A phylogenetic tree constructed with the protein sequences of GRD, LCCH3, RDL, 8916, α-like, and GABA3 subunits of the selected organisms revealed that GABA receptor proteins are segregated into five clades: the four well-known clades (RDL, LCCH3, GRD and 8916) and a fifth clade containing the sequences of γ-aminobutyric-acid receptor Phh-α-2, Pa-8916_2, Bg-8916_2, Ls-α-like, Go-GABA3, Ir-GABA3 and Pt-GABA3 (Figure 2).

Moreover, arthropods 8916, GRD and the new (fifth) clade showed a close phylogenetic relationship, suggesting the possibility of having a common ancestor. Considering these results, we hypothesized the existence of a new GABA receptor subunit in arthropods that we named HoCas for Homologous to Cys-loop α-like subunit. At the sequence identity level, insect HoCas share identity from 37.2% to 62.7% whereas Chelicerata-HoCas share identity from 49.9% to 62.7% (Table 3). Inside insects, Phh-HoCas is more closely related to Bg-8916_2 with 39.5% sequence identities.

**Figure 3:**
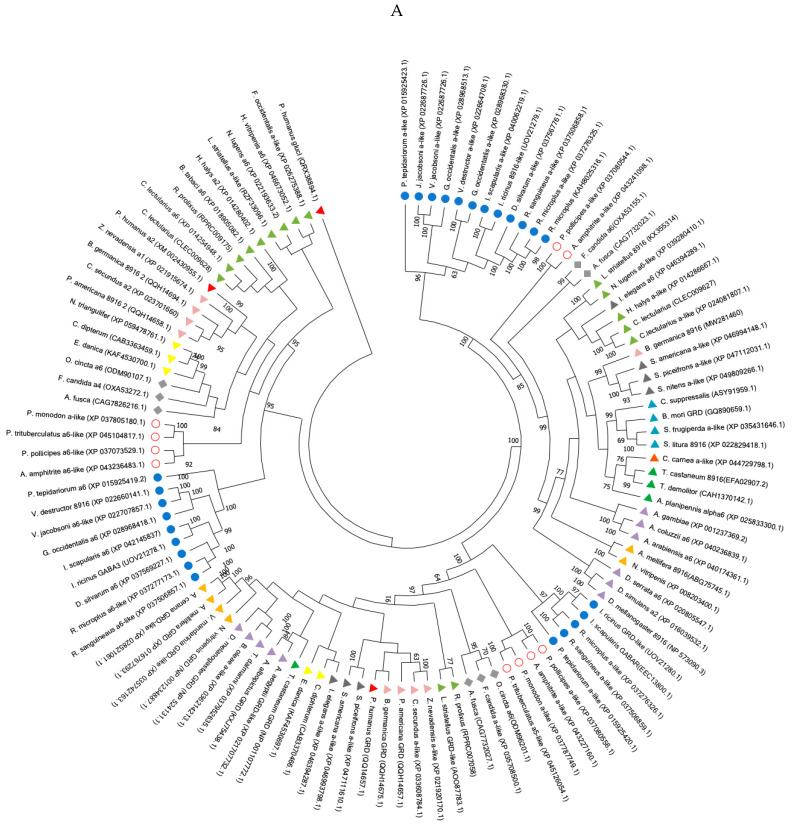

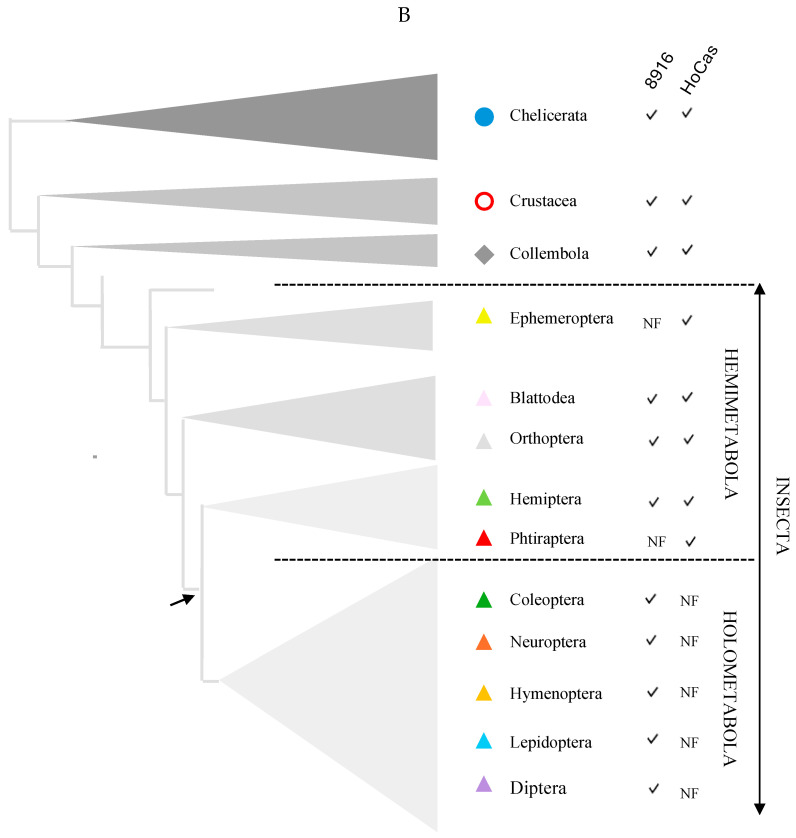
(**A**). Phylogenetic analysis of GRD, HoCas, and 8916 subunits in different arthropods; neighbour-joining phylogenetic tree of GRD, 8916, and HoCas subunits. The phylogeny includes sequences from Chelicerata, Crustacea, Collembola, and representative insects. Phh-GluCl was used to root the tree. (**B**). Schematic representation showing the presence of 8916 and HoCas in Arthropodes. The tree is adapted from [39]. NF: not found.

In order to confirm the existence of this new clade/subunit, using BLASTP, we compared the protein sequences of Phh-HoCas, Am-8916 and Phh-GRD as templates against different insect orders, namely Crustacea, Collembola, and Chelicerata. Firstly, our results confirmed the existence of the three separate clades (GRD, 8916, and HoCas) (Figure 3A). Secondly, 8916 is conserved among arthropods as sequences were present in Chelicerata, Collembola, Crustacea, and most of the tested insect orders (Blattodae, Orthoptera, Hemiptera, Coleoptera, Neuroptera, Hymenoptera, Lepidoptera, and Diptera (Figure 3B). A systematic search for the 8916-encoding gene in the genome of *Pediculus humanus* (Phthiraptera) ended up with no results, suggesting that the human body louse possesses the four GABA subunit genes: *Phh-grd*, *Phh-rdl*, *Phh-lcch3*, and *Phh-hocas,* but possibly misses *Phh-8916* (Figure 3). Finally, the sequences of the newly described clade “HoCas” were found in Chelicerata, Crustacea, Collembola, and Hemimetabola, but not in Holometabola (Figure 3B). Inside each clade (GRD, 8916, HoCas), the sequences of Insecta, Collembola, Crustacea, and Chelicerata are well segregated (Figure 3A). Interestingly, *Bombyx mori* Bm-GRD clustered with other insect 8916 genes (Figure 3A), confirming that, as previously described, the Bm-GRD sequence deposited in NCBI (Accession numbers: GQ890659.1 or NP_001182633.1) may be the Bm-8916 gene [38].

### 3.2. Cloning and Sequence Analysis of Phh-Hocas

We characterized the *Phh-hocas* mRNA ends by 5′ and 3′ RACE-PCR on total RNA extracted from nits by using set of primers binding in exon 2 and in exon 6, respectively (Table 1; Figure 1A). For 5′ RACE PCR, we identified one TSS 38 nt upstream from the starting codon, and for 3′ RACE two 3′ end sites located, respectively, 146 nt and 68 nt downstream from an ATTAAA/AT polyA signal (Figure 1A). Reverse transcription PCR (RT-PCR) on total RNA extracted from lice with the pair of primers F3-R3 and F3-R4 allowed us to amplify 3 transcripts (Figure 1B). The sequences of *Phh-hocas* transcripts were deposited in GenBank with the corresponding accession numbers: the transcript *Phh-hocas-1* (OQ831857) confirmed the in silico annotation, the second transcript *Phh-hocas-2* (OQ851502) resulted from a partial retention of intron 5, and the third transcript *Phh-hocas-3* (OQ851503) resulted from alternative splicing of exon 5 and ended early at the beginning of E7. Translation and analysis of the encoded proteins revealed that both *Phh-hocas-2* (with a premature stop codon in the retained intron) and *Phh-hocas-3* code for truncated proteins missing TM4. Finally, the full-length cDNA of *Phh-hocas-1* is 1819 bp, with an ORF of 1713 bp encoding a protein of 570 amino acids.

**Figure 4:**
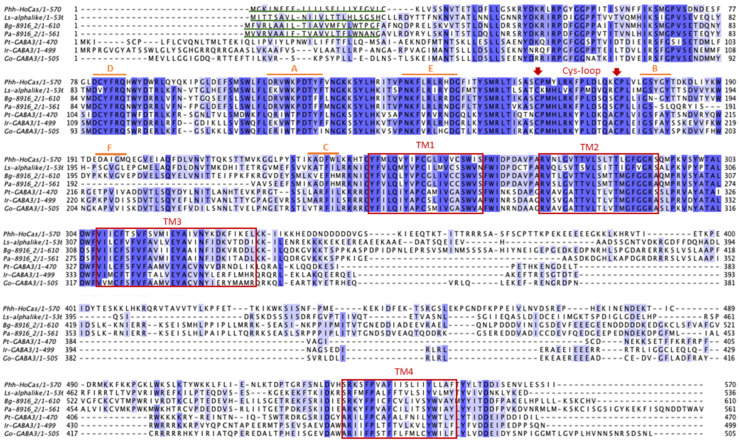
Multiple sequence alignment of the deduced amino acid sequence of Phh-HoCas subunit with related sequences from other species. GenBank accession numbers are as follow: Ls-alphalike (RZF33096.1), Bg-8916_2 (QQH14694.1)*,* Pam8916_2 (QQH14658.1), Pt-GABA3 (XP_015925419.1). Ir-GABA3 (UOV21278), Go-GABA-3 (XP_028968418.1). Signal peptides are underlined in green and the four transmembrane domains (TM 1-4) are indicated by red boxes. The cysteine forming the cys-loop are highlighted with red arrows. The six loops (A-F) involved in binding to the natural ligand are indicated by orange lines.

The multiple sequence alignment of HoCas in Insecta and Chelicerata revealed that they possess all the typical features of cys-loop LGICs: the two cysteines, Phh-HoCas-C^161^ and Phh-HoCas-C^175^, characteristic of the cys loop motif; 4 TM domains, TM1 (Phh-HoCas-Y^242^-S^261^), TM2 (Phh-HoCas-R^272^-S^292^), TM3 (Phh-HoCas-V^307^-L^334^), and TM4 (Phh-HoCas-S^533^-F^552^); the six loops (A-F) involved in binding to the natural ligand; and a signal peptide at position 1 to 23 (Figure 4).

### 3.3. Spatial and Temporal Expression of GABA Receptor Subunits

**Figure 5:**
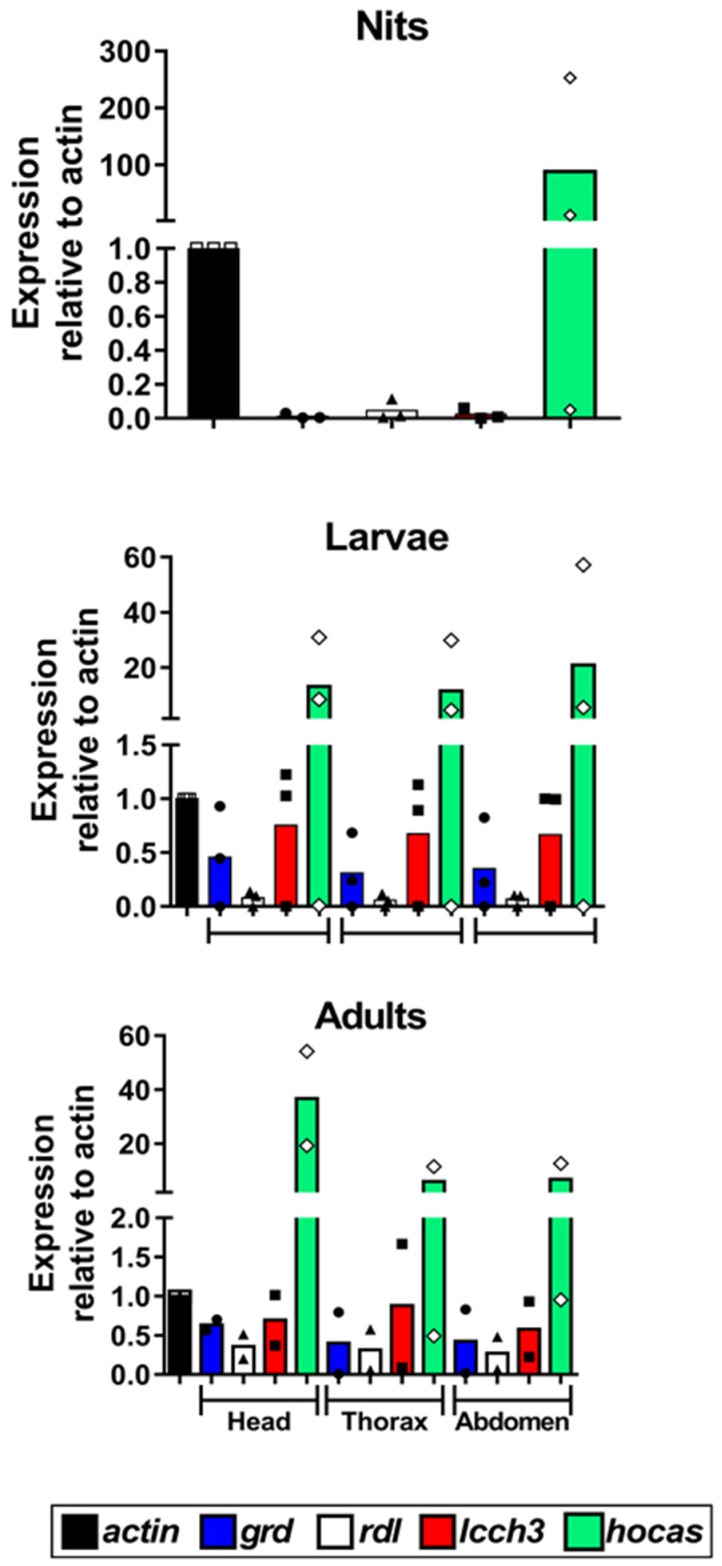
Relative expression of *Phh-hocas, Phh-grd, Phh-rdl,* and *Phh-lcch3* throughout the development stages and in different parts of the human body louse. qPCR applying comparative cycle threshold experiment (2^^−ΔCt^) was performed. Data presented as median and normalized to the expression of *actin* as endogenous control. Statistical difference (*p* value ≥ 0.05) was calculated using ANOVA followed by Tukey’s multiple comparisons test. The results of *Phh-grd*, *Phh-rdl*, and *Phh-lcch3* have been already published without the results of *Phh-hocas*, analyzed at the same time [30].

In order to verify the expression of *Phh-hocas* in the human louse, we performed qPCR experiments with RNA extracted throughout the development stages (nits; L1, L2, L3 larval stages; and adults) and from different parts (head, thorax, and abdomen) (Figure 5). *Phh-hocas* was found to be expressed in all parts of adult body louse (Figure 5 bottom) and throughout all development stages (Figure 5 top and center). Relative to *actin*, the expression of *Phh-hocas* is highest in nits (about 100-fold) (Figure 5 top) followed by larval stages (20-fold) (Figure 5). In adults, relative to *actin*, the highest expression of *Phh-hocas* was in the head (40-fold) then in the thorax and abdomen (about 10-fold) (Figure 5 bottom). Furthermore, *Phh-hocas* was always much more highly expressed than the other subunits.

## 4. Discussion

GABA is the main inhibitory neurotransmitter in insects targeted by insecticides. In insects, four GABA subunits segregated in 4 separate clades are well described: RDL, GRD, LCCH3, and 8916. Depending on their association to formulate functional receptors, they could have distinct sensitivities to the natural ligand and different pharmacological profiles [13,17,20,22,23,27,28].

In the present work, in addition to our previous studies describing three GABA subunits in the human body louse, *Phh-rdl* [31], *Phh-grd*, and *Phh-lcch3* [30], we have now identified another gene encoding for a putative GABA receptor subunit that we named *Phh-hocas* for Homologous to Cys-loop α like subunit. Phh-HoCas formed a separate clade distinct from the well-known GRD, LCCH3, RDL, and 8916 subunits. In this clade, Phh-HoCas clusters with the non-classified Bg-8916_2, Pa-8916_2, Ir-GABA-3, Go-GABA3, Pt-GABA3, and Ls-α-like sequences, suggesting the existence of a new (fifth) GABA receptor clade in arthropods.

Our systematic search revealed that HoCas and 8916 subunits are present in Insecta, Collembola, Crustacean, and Chelicerata and share a common ancestor with GRD subunits who lived about 600 million years ago [39,40]. It has been shown that, in the genome of *A. mellifera, D. melanogaster,* and *T. castaneum,* the *8916* gene is located close to *lcch3*, while in the genome of *Pediculus humanus, Phh-hocas* is close to *Phh-lcch3.* Interestingly, our search revealed that in the genomes of Hemiptera, Chelicerata, and Blattodae *8916*, *hocas* and *lcch3* genes are located close to each other, suggesting that these genes originated from three duplication events: the first duplication event from the ancestral gene A1 leading to LCCH3 and the ancestral gene A2, A2 was further duplicated in HoCas and the ancestral gene A3, which finally duplicated to give 8916 and GRD (Figure 2). However, the absence of *hocas* in all Holometabola (Figure 3B) is intriguing. If a common ancestor of *hocas* existed as suggested previously, one hypothesis would be the loss of this gene during speciation event of Holometabola sub-order [40].

In the genome of *P. humanus humanus*, our analysis failed to identify the gene encoding for 8916. Assembly of insect genomes is challenging due to the large stretch of AT; we cannot exclude defaults in assembly of the human louse genome, as revealed by the missed sequences found in the *Phh-GRD* genomic annotation and the strikingly very long *Phh-GRD* gene (>7 kb) encompassing 3 contigs [30]. Confirmation of the genomic annotation by comparing with the transcriptomics data is a good method to have a complete view of cys-loop LGICs as observed in *I. ricinus* and *I. scapularis* [32]. In the human body louse, transcriptomics analysis [41] validated the genomic annotation [36] confirming the absence of *Phh-8916*. The obligatory parasitism status of *P. humanus* could explain the loss of some genes like *8916* in favor of others, similar to what described in *Acyrtosyphon pisum* lacking *grd* and *lcch3* but having a second *GluCl* subunit [42].

In this study, we cloned the complete cDNA sequence of *Phh-hocas* of *Pediculus humanus humanus* (Phthiraptera) using qPCR and RACE-PCR techniques. Moreover, besides the full-length transcript, we identified two transcript variants of *Phh-hocas* resulting from a partial intron 5 retention/alternative splicing and premature polyA signal, both encoding proteins missing TM4. The possible role of these truncated variants is not clear and requires more investigation. Alternative splicing and retention of introns in the genes encoding cys-loop LGICs have been described in many insects and result in proteins with variable functionalities and sensitivities to insecticides. For example, retentions located at the intracellular domain between TM3 and TM4 have been observed in *varroa destructor* (Vd-GRD), leading to marked changes in GABA sensitivities compared to variant without retentions [27].

Results of the relative expressions revealed a remarkable higher expression of *Phh-HoCas* compared to other subunits in all tested parts of human body louse and throughout the development stage, while the expression of *Phh-grd, Phh-lcch3* and *Phh-rdl* is almost the same, raising a question about the possible physiological role of *Phh-hocas*. It would be interesting to see if similar expression profiles can be obtained in other species and to test the effect of inhibition of gene expression in the louse physiology using RNAi. Since *Phh-hocas* was found to be expressed at higher levels compared to other GABA subunits of human louse especially in nits, it remains to determine whether Phh-HoCas is able to reconstitute homo or hetero pentameric functional GABA receptor. If so, and hence most of the commonly used pediculicides are not active against nits, Phh-HoCas could be a potential target to design a novel pediculicides against adult louse and nits.

## Figures and Tables

**Table 1:** Primers used to characterize the 5′ and 3′ends of *Phh-hocas* by RACE-PCR and to amplify full-length cDNA.

Primer	Sequence (5′-3′)
5′ RACE first PCR (R1)	ACGCCAATCATACCAATGTTGTCG
5′ RACE nested PCR (R2)	GTTATCCGATACGGGTCCCATACT
3′ RACE first PCR (F1)	CCGGAAATAGTATTAAACGTTGATTCTC
3′ RACE nested PCR (F2)	GAGACGTACATTCTAGAAAAAGTTTTCC
Full length forward (F3)	GGCTTTAGTGAACAAAACAAATGG
Full length reverse (R3)	TTAAATGATAGAAGACTCTAAAAC
Full length reverse (R4)	CGATTCTGTATAATCAATTTCCGG

**Table 2:** Primer pairs designed for qPCR for *Phh-hocas*, *Phh-grd*, *Phh-rdl, Phh-lcch3,* and *Phh-β-actin*.

Primer	Sequence (5′-3′)	Product Size (Amplicon)
*Phh-hocas*-Forward	CCGGAAATTGATTATACAGAATCG	173 bp
*Phh-hocas*-Reverse	CCGGAAATAGTATTAAACGTTGATTCTC	
*Phh-grd*-Forward	GGTTTGGAAGCAAGAACGGAC	155 bp
*Phh-grd*-Reverse	CCGAAATAACATTCACCCGAACCG	
*Phh-rdl*-Forward	GCGAAAAAGTAGATTTATGGCG	174 bp
*Phh-rdl*-Reverse	GTACCTCCTTTGGAATGAGC	
*Phh-lcch3*-Forward	GGGTATAACCACGGTACTAAC	171 bp
*Phh-lcch3*- Reverse	CTTGCTCCCCAATATGTATAG	
*Phh-β-actin*-Forward	TGCCACATGCTATTCTCCGT	60 bp
*Phh-β-actin*-Reverse	TTCATTCACTACCACTGCCG	

**Table 3:** Percentage identity among the amino acid sequences of Phh-HoCas, GABA-3, and α-like GABA receptor subunits from insects (Pa for *P. americana*, Bg for *B. germanica,* Ls for *Laodelphax striatellus,* and *Phh* for *Pediculus humanus humanus*) and arachnids (Pt for *P. tepidariorum,* Ir for *Ixodes ricinus*, and Go for *Galendromus occidentalis*).

	Pt-GABA3	Ir-GABA3	Go-GABA3	Bg-8916_2	Pa-8916_2	Ls-Alike	Phh-HoCas
Pt-GABA3		49.9%	50.4%	31.6%	29.4%	32.1%	31.1%
Ir-GABA3	49.9%		62.7%	32.4%	30.2%	31.2%	29.8%
Go-GABA3	50.4%	62.7%		30.7%	28.1%	30.1%	28.4%
Bg-8916_2	31.6%	32.4%	30.7%		62.7%	42.1%	39.5%
Pa-8916_2	29.4%	30.2%	28.1%	62.7%		38.5%	37.2%
Ls-alike	32.1%	31.2%	30.1%	42.1%	38.5%		37.5%
Phh-HoCas	30.1%	29.8%	28.4%	39.5%	37.2%	37.5%	

## Data Availability

The original contributions presented in the study are included in the article, further inquiries can be directed to the corresponding author.
